# Delivery of a community-based peer mentorship program for people with spinal cord injury at a rehabilitation center

**DOI:** 10.3389/fresc.2023.1296505

**Published:** 2023-11-29

**Authors:** Zhiyang Shi, Jacques Comeau, Gordon A. Bloom, Heather Gainforth, Aliki Thomas, Shane N. Sweet

**Affiliations:** ^1^ Department of Kinesiology and Physical Education, McGill University, Montreal, QC, Canada; ^2^ Centre for Interdisciplinary Rehabilitation Research in Metropolitan Montreal, Montreal, QC, Canada; ^3^ School of Health and Exercise Sciences, University of British Columbia Okanagan, Kelowna, BC, Canada; ^4^ International Collaboration on Repair Discoveries (ICORD), University of British Columbia, Vancouver, BC, Canada; ^5^ School of Physical and Occupational Therapy, McGill University, Montreal, QC, Canada

**Keywords:** spinal cord injury, rehabilitation, community services, peer support, qualitative research

## Abstract

**Introduction:**

Community-based spinal cord injury (SCI) organizations deliver peer mentorship programs in rehabilitation settings. Little is known on how these programs are delivered through the collaboration between community-based SCI organizations and rehabilitation institutions. This study aimed to identify barriers, facilitators, and collaboration processes within a SCI peer mentorship program provided by a community-based organization at a rehabilitation center.

**Methods:**

A qualitative case study design was applied. Seven participants were recruited, including two mentees, two mentors, one program director of the community-based SCI organization, and two healthcare professionals of the rehabilitation center. Each participant completed a one-on-one interview. Data were analyzed inductively and deductively based on the Consolidated Framework for Implementation Research (CFIR).

**Results:**

Ten factors were identified to influence the delivery of the peer mentorship program, including nine CFIR constructs. Successful delivery of the program required strong, collaborative inter-professional relationships between health professionals and community organizational staff (e.g., peer mentors) as facilitators; whereas potential cost, minimal patient needs, and limited mentor resources were found to be barriers. Engaging health professionals by initiating communications, reflecting and evaluating the program collectively with health professionals were important collaboration processes for the community-based organization to maintain effective partnership with the rehabilitation center.

**Discussion:**

The collaboration processes and strategies to addressing/leveraging the barriers and facilitators may inform evidence-based practice to establish and optimize the delivery of SCI peer mentorship programs in various rehabilitation settings.

## Introduction

Spinal cord injury (SCI) refers to any damage or lesion to the spinal cord that results in autonomic, motor, and sensory impairments and lifelong disability. After an SCI, people often begin a rehabilitation process in which they experience significant adjustment to life (1). One strategy that has been utilized to support the rehabilitation and community re-integration for people with SCI is peer mentorship (2). Peer mentorship is a form of peer interaction aiming to help individuals who share similar lived experiences adapt and thrive (3, 4). In Canada, provincial community-based SCI organizations collaborate with more than 41 hospitals and rehabilitation centers to make peer mentorship available for many Canadians with SCI (5).

Delivering peer mentorship programs in rehabilitation settings often relies on collaborations between community-based SCI organizations and rehabilitation institutions (6). Peer mentorship literature has mostly gathered insights from mentors, mentees, family members, and community organizational staff to understand characteristics and outcomes of peer mentorship programs (5, 7). However, it remains unclear how peer mentorship programs are delivered through collaborations between community-based SCI organizations and rehabilitation institutions. Additionally, the role of health professionals and their relationships with community organizational staff (e.g., peer mentors) within peer mentorship programs largely remains unknown. Without this knowledge, it is difficult to optimize the implementation of SCI peer mentorship programs within rehabilitation contexts.

Some international studies examined the integration of SCI peer mentorship programs into rehabilitation settings (8–14). For one, Cabigon et al. (2019) investigated the inter-professional collaboration between peer mentors and health professionals in delivering SCI bowel education and demonstrated the feasibility of the program at an American rehabilitation center (8). In addition, a Danish study described the process of health professionals recruiting and training peer mentors prior to the delivery of a SCI peer mentorship program (11). These two studies highlighted that the collaborative relationship between SCI peer mentors and health professionals was important to the programs. However, they focused on the development phase of the peer mentorship programs without investigating how SCI peer mentors and healthcare professionals collaborate to maintain SCI peer mentorship programs.

Theoretical frameworks in implementation science may help us understand the collaboration between rehabilitation institutions and community-based SCI organizations in delivering peer mentorship programs (15). One framework that was specifically designed for investigating the implementation and delivery of a program/service is the Consolidated Framework for Implementation Research (CFIR) (16). The CFIR organizes 39 factors (e.g., networks and communications) that influence the implementation of a program into five domains (e.g., intervention characteristics). The CFIR has been used to investigate programs/services for people with SCI (17) and allowed the researchers to examine various aspects of the programs, including relationships among the personnel involved (15, 18).

The purpose of this study was to identify barriers, facilitators, and collaboration processes within a SCI peer mentorship program provided by a community-based organization at a rehabilitation center. Framed around the CFIR, three main research questions were: (1) how was the peer mentorship program delivered through the collaborations between the community-based organization and the rehabilitation center; (2) what were the barriers and facilitators to the delivery of the program; and (3) what were the inter-professional relationships between the community organizational staff (e.g., SCI peer mentors) and the rehabilitation professionals?

## Methods

### Design

We applied a qualitative case study design (19), which allowed us to collect contextual information on the program and investigate how the peer mentorship program was delivered (19, 20). We situated this study within a post-positivist paradigm (21) and assumed that an external reality existed independent of our knowledge of it (i.e., modified realist ontology). Our research team consists of one retired SCI peer mentor (JC) and five researchers (AT, GB, HG, SS, and ZS) who self-identify as being non-disabled. AT, GB, HG, and SS are associate/full university professors. ZS is a senior doctoral candidate with seven years of research experience, primarily using qualitative methodologies. AT and HG have expertise in the field of implementation science/knowledge translation within the rehabilitation and disability contexts. GB, HG, SS, and ZS conducted multiple research studies on peer mentorship in various contexts, such as parasport and SCI. HG, SS, and ZS have a research focus on social participation and well-being promotion among individuals with SCI. GB is an expert in qualitative research who assisted JC, SS and ZS to critically think about the data. This combination of the diverse expertise resonates with the focus and design of the current study. Our different knowledge backgrounds and research experiences inescapably shaped how we formulated the research questions and interpreted different aspects of the SCI peer mentorship program [i.e., subjectivist epistemology; (21)].

### Setting

We identified a local community-based SCI organization that offers peer mentorship programs in both the community and rehabilitation settings, including a rehabilitation center that provides services to individuals with SCI. There is no cost to patients in the rehabilitation center to participate in the peer mentorship program. The community-based organizational staff, including SCI peer mentors, are on-site at the rehabilitation center and work directly with a multidisciplinary healthcare team including occupational therapists and physical therapists. The peer mentorship is mentee-focused in that topics of the conversations can vary depending on mentees' specific needs. Peer mentorship is delivered through both (a) informal, unstructured conversations between mentors and mentees, which can happen at bedside or common areas (e.g., cafeteria) at the rehabilitation center and (b) formal, structured conversations either by information sessions delivered by mentors and rehabilitation staff or individuals, topic-focused discussion with a mentee. The mentorship relationship can also continue after in-patient rehabilitation process as mentors also provide mentorship to people with SCI living in the community. We chose to look at this program because it is recognized as long-standing and successful, with more than forty years of continuous delivery.

### Participants and data collection

We recruited a purposive sample of seven participants from the community-based SCI organization and the rehabilitation center. Participants were individuals involved in the peer mentorship program with different roles, including two mentees, two mentors, one program director of the community-based SCI organization, and two healthcare professionals of the rehabilitation center (one social worker and one kinesiologist). The sample size aligned with the qualitative case study design (22). All seven participants were adults, had no cognitive impairments, and were able to communicate in English or French. Eligible healthcare professionals must have experience of interacting with a SCI peer mentor(s) during the last two years. Eligible peer mentees and mentors must have engaged in the peer mentorship program at the rehabilitation center during the last two years. This study was approved by our university research ethics board.

We provided the information on this study (e.g., purpose, research questions, procedures) and obtained participants' consent using an online consent form embedded in emails. Each participant completed a virtual, one-on-one, audio-recorded interview with the first author (ZS) using a semi-structured interview guide. The interview guide included questions selected from the CFIR interview guide tool (cfirguide.org/) and the questions were adapted to the different roles of the participants (Supplementary Appendix A). For example, the question “How do you feel about the intervention being used in your setting?” (CFIR construct: Knowledge and beliefs about the intervention) was adapted to “How do you feel about the peer mentorship program at the rehabilitation center?”. Each interview was planned to be completed within one hour.

### Data analysis

All seven interviews were transcribed verbatim, resulting in 128 pages of text. Transcripts were analyzed using a two-step (i.e., deductive and inductive) analytical approach (23). Deductively, participants' quotes that were found to be relevant to any of the 39 CFIR constructs were coded with the constructs names. Inductively, data that did not align with CFIR constructs but were relevant to our research questions were coded with a non-CFIR construct. To represent broader ideas identified within the data, all deductive and inductive constructs were examined and organized into overarching themes. The first author (ZS) conducted the deductive and inductive coding using Nvivo and had multiple discussions with the co-authors to develop the themes. Specifically, ZS coded and extracted the data relevant to the research questions using CFIR. The development of themes was an iterative process, in which ZS had multiple meetings with JC to identify the initial themes. These initial themes were then critically examined with SS, resulting in modification of the initial themes. Next, GB helped re-organize the themes and enhance the clarity in reporting the results as a critical friend. This two-step approach allowed us to identify elements relevant to the peer mentorship program based on CFIR, while also exploring information beyond CFIR constructs. Because participants had different roles in the program, interview data were first analyzed within the same type of participants (e.g., mentees) and then across the different types of the participants (e.g., mentees vs. mentors) to identify common themes (19).

### Study quality

We ensured the quality of this study following the eight universal criteria named by Tracy (2010) (24), including (1) worthy topic: by clearly defining the research purpose and highlighting its relevancy to the SCI population; (2) rich rigor: by adopting the CFIR to guide the data collection and analysis; (3) sincerity: by engaging critical friends in the analysis and recognizing our subjective values influencing the interpretations; (4) credibility: by spending time building rapport with participants during the interviews and involving the author with lived experience (JC) in the data analysis. JC lives with SCI and had worked as a SCI peer mentor for over ten years. JC's input ensured the themes identified were relevant to the delivery of the peer mentorship program from their perspective; (5) resonance: by incorporating participants' quotes into the results; (6) significant contribution: by highlighting the study implications to the SCI literature and rehabilitation practice (7) ethics: by following the procedures approved by the university ethics board; and (8) meaningful coherence: by applying research methods aligning with the qualitative case study design (25, 26). A COnsolidated criteria for REporting Qualitative Research (COREQ) checklist was attached (Supplementary Appendix B) (27).

## Results

**Figure 1:**
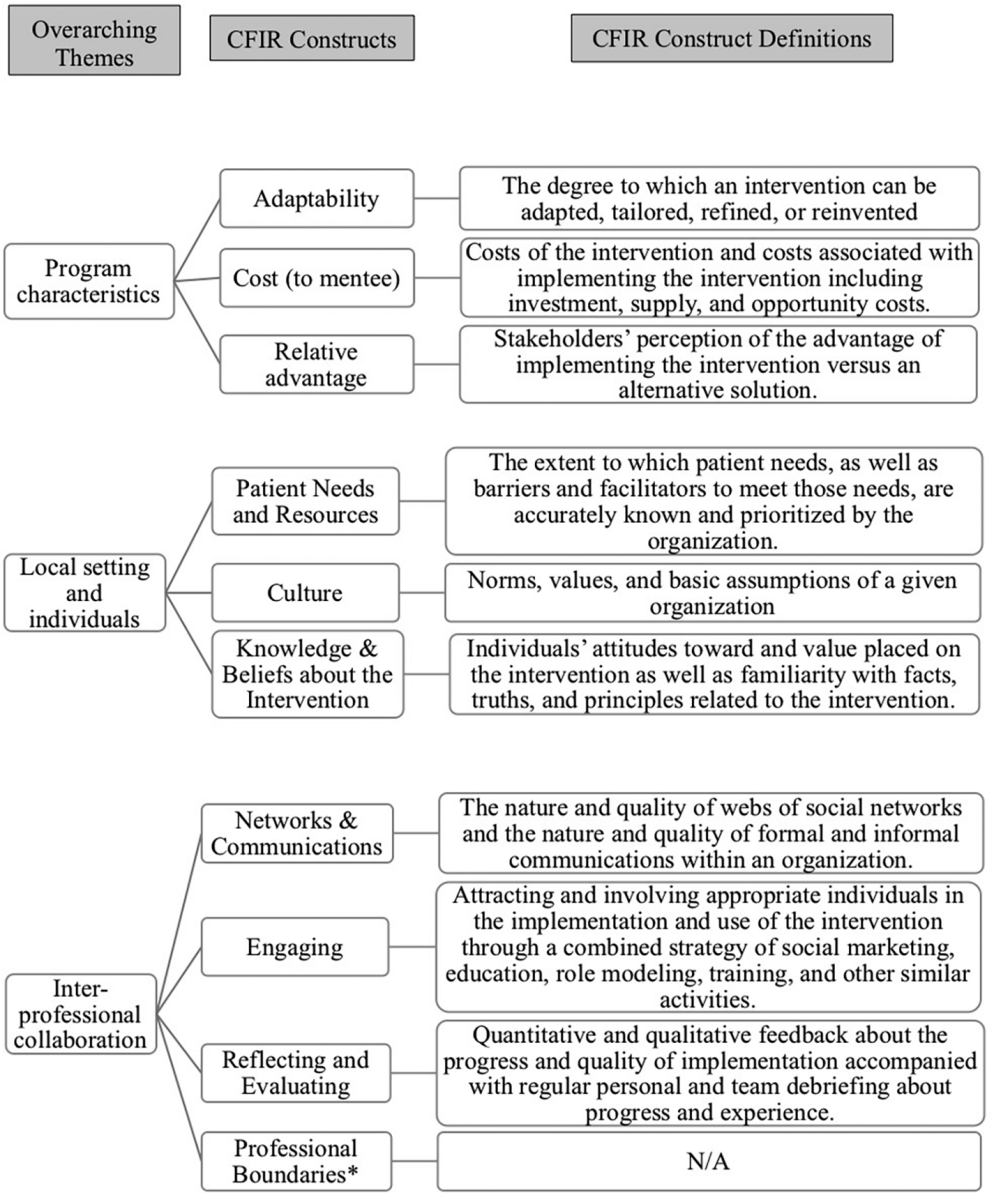
Overarching themes and CFIR constructs with its definition. * Inductive themes identified.

The data were organized into three overarching themes: program characteristics, local setting and individuals, and inter-professional collaboration. These overarching themes included ten of 39 CFIR constructs identified in the deductive analysis and one inductive, non-CFIR construct (marked with *). Figure 1 summarized the organization of the overarching themes and constructs. We adapted the names of some CFIR constructs (e.g., cost to mentees) to ensure fit in the local context and the delivery of the peer mentorship program. Participant quotes were also presented in the results.

### Program characteristics

Adaptability, cost, and relative advantage were three characteristics of the peer mentorship program that were identified to influence the delivery of the program at the rehabilitation center.

Having a variety of methods to deliver the peer mentorship program enables the community-based organization and the rehabilitation center to adapt, tailor, refine, or reinvent the program as needed [Adaptability]. The rehabilitation center and the community-based organization typically deliver the program by creating an environment where mentors and patients interact through informal conversations. However, they also offer mentees a regular magazine, group-based coffee meetings, and a series of courses on SCI, which allows mentees to interact with peer mentorship resources that meet their needs. Furthermore, the community-based organization and the rehabilitation center made adaptations to the program during the COVID-19 pandemic by coordinating formal in-person, one-on-one meetings between mentors and mentees to maintain the delivery of the program.

“Normally we were able to do a lot of activities in-house, at the [rehabilitation center], but we couldn’t do any for a long time [during the pandemic]. The patients were [restricted] in their rooms… That's when we realized that it was so complicated to meet with the patients. We asked the nurse on the second floor to help us plan formal meetings in a local area with each patient with spinal cord injury. As I said, if we don’t do that, I would say we’re going to lose so many people, (and) we can’t do that. So, it's the way we adapted to the new situation.” —Julie (program director of the community-based organization)

Despite the fact that the current peer mentorship program is free for patients with SCI at the rehabilitation center, any potential monetary cost to access the program can become a possible barrier for patients with SCI to participate in the program [Cost to mentee], as Jack (mentee) said:

“You can get all the information on the Internet. Anyways, you know? … I can read all over the Internet and there's forums, you know, there's Christopher Reeves Foundation. There's all kinds of stuff where I can talk to many experienced paraplegics on the Internet. Yeah, so for me, there's no point in paying for [peer mentorship] at all… If they were to charge people for their services, I would not be… I’d rather pay for medical service.”

Finally, the peer mentorship program was found to be a valuable addition to regular rehabilitation services [Relative advantage]. The health professionals noted the peer mentors were able to provide disability-specific tips and helpful suggestions based on their lived experience with SCI. These suggestions helped the health professionals supplement their medical and therapeutic recommendations. As Anna (social worker) noted:

“We are professionals, we didn’t go through that [living with SCI]. They [mentors] really lived the situation, because some patients will say [to us] ‘you didn’t live it’ and they are right, we are here to accompany, we didn’t really live it, whereas the mentors have really lived the situations.”

### Local setting and individuals

The delivery of the peer mentorship program related to three CFIR constructs, focusing on patients' needs, a positive organizational culture within the local rehabilitation center, and health professionals' adequate knowledge and beliefs about peer mentorship.

A strong need for peer mentorship identified among the SCI patient clientele at the rehabilitation center appears to facilitate the delivery of the program. Because the community-based organization partners with multiple hospitals and rehabilitation centers in the region, it tends to allocate mentor resources and prioritize institutions with a larger SCI clientele and/or a greater patient need for the peer mentorship program [Patient Needs and Resources]:

“In most settings, they don’t have a lot of people with SCI there. And there are no centers across our province other than the [rehabilitation center] where patients are onsite with a [SCI] group… We know that there's always 20 patients, so we have people in house all the time because we’re going to be crossing and seeing people at the center where you go in once or twice a week. You can’t hire somebody who's going to work two hours a week or three hours a week and say, ‘Be there on Tuesday from three to four and Thursday from ten to eleven.’ In those situations, we tend to typically offer services more personalized where somebody will talk over the phone, you know, when you’re interested in talking. But having somebody on site is not always feasible.” — Jean (mentor)

Second, an organizational culture that values new changes and is willing to adapt to changes at the rehabilitation center was another facilitator for the delivery of the peer mentorship program [Culture]. In the current peer mentorship program, the program director of the community-based organization had experienced different organizational cultures within the rehabilitation center by working with health professionals over many years. She highlighted the impact of a recent change in staff that created a more positive organizational culture and resulted in improved outcomes of the peer mentorship program.

“People who retired and the new people who came in found that there was a culture change. It's really healthier. We’re giving a very positive input to the patients.”—Julie (program director of the community-based organization)

Third, the positive attitudes toward and the value placed on the peer mentorship program by the health professionals were identified as key facilitators to the delivery of the program [Knowledge and Beliefs about the Intervention]. The ongoing interactions with the mentors helped the health professionals expand their knowledge on SCI peer mentorship and thus develop a strong commitment to collaborating with the mentors.

“We learn a lot from their [mentors] experience…If we need to realign our thinking, our vision, it's always a question we ask [mentors], ‘what you had wanted to change in rehabilitation, what we could have done better, and how you would have liked it if we had talked about [certain] things.’ To have this feedback from them [mentors] is very important for us to be able to align our work and to be in the right direction.” —Nada (kinesiologist)

### Interprofessional collaboration

The peer mentorship program requires a cohesive interprofessional collaboration that consists of engaging the health professionals in the program, as well as evaluating and reflecting on the delivery of the program. Interprofessional collaboration can also be built by establishing strong communication channels and clear boundaries between the health professionals and the community-based organizational staff, particularly the mentors.

One mechanism that appeared to build inter-professional collaboration is having quality social networks and communications between the mentors and the health professionals [Networks & Communications]. In this case, the peer mentors have an office at the rehabilitation center and share workspace with the health professionals. This proximity creates opportunities for frequent, informal communications between the health professionals and the mentors, while facilitating resolution of misunderstandings around patient care:

“The mentors are on the same floor as us, they are practically in our offices so we get to see them. It's [the communication] quick, it's easy, it's efficient. They are also often around the floor for a variety of reasons, so we can interact with them as they pass… Sometimes it [the communication] can be informal, like in the hallway and we bump into a member of [community-based organization]. Sometimes something happens where they [mentors] might have made a suggestion, such as about an adjustment or type of wheelchair, but as we know the client, and our recommendation might be a reason that's not so obvious to someone else, so we might have to talk about why we suggested what we did vs. what they thought.” —Nada (kinesiologist).

Another mechanism that the community-based organization strengthens the interprofessional collaboration is attracting and involving the health professionals in the peer mentorship program by helping them understand mentors' roles and benefits of the peer mentorship program [Engaging]:

“They [health professionals] have to know the [SCI] organization well, the [healthcare] team has to understand the importance of that [mentorship]. It must be well explained to the teams that are giving the care: what is the role of a peer mentor and what they bring to people. You [health professionals] really must understand that as a base. Once they understand that, they’re going to be more motivated to put in place a service like [peer mentorship], and they’re going to be able to see how it can help them in their interventions.” —Julie (program director of the community-based organization)

Consistent evaluation and reflection on the progress and quality of the peer mentorship program was another important process of the interprofessional collaboration [Reflecting and Evaluating]. Within the current program, the program director of the community-based organization has been taking an integral role in tracking the progress and quality of the peer mentorship program. However, a team approach through collaborating with the health professionals and the mentors is needed due to emerging challenges in delivering the program. As these two quotes below demonstrated,

“I do all the budgeting, I hire the people, all the work of a manager… I make sure that they [mentors] are present at the [rehabilitation center]. I make sure that as much as possible we meet all the people who come to the [rehabilitation center], obviously those with an SCI… Of course, when there are new employees, new senior integration mentors, I make sure that the [rehabilitation center's] management is aware of this, that they [the mentors] have training… We are challenged in different ways. We arrive and sometimes the clientele is really older so we have to adapt our intervention a little bit, the activities we offer to succeed in getting people interested.” —Julie (program director of the community-based organization)

“I think we [the health professionals and the community-based organizational staff] could have even more discussions [on letting patients participate in the peer mentorship program]. I think sometimes there might be disagreement between us [the health professionals] and the mentors, [because] in the rehabilitation environment where we [the health professionals] have to be a little more careful with new spinal cord injuries [patients]. Sometimes [the health professionals believe] they are not ready to be sent to an activity of [the community-based organization] because they may not have someone to help them transfer.” —Nada (kinesiologist)

While the interprofessional collaboration is key to the peer mentorship program, maintaining professional boundaries between the health professionals and the community-based organizational staff is also important [Professional Boundaries*]. As Betty (mentor) mentioned, “We are not registered in their rehabilitation program. We are completely independent. Yes, we collaborate with the health specialists, but we remain an independent entity”. In this case, the rehabilitation center and the community-based organization have an agreement that explicitly outlines the boundaries regarding the mentors' access to patient confidential information. Although the agreement does not include all aspects of the mentors' responsibilities, it helps the health professionals and the mentors understand and adhere to their roles in patient care:

“We [mentors] must not interfere with the role of social workers in the rehabilitation center. For example, an occupational therapist should not feel challenged in what she does in comparison to a senior mentor in the center. If this happens, we have to resolve the situation. Everyone has to know their place. So that's really important… We never directly give the patient the clinical judgment, that's being a professional.” —Julie (program director of the community-based organization)

Although the professional boundaries were clear to the mentors and the health professionals, these boundaries might be blurry for patients. Patients may expect clinical guidance from mentors and can potentially create difficult situations during their interactions with mentors and/or health professionals, as Frank (mentee) said: “I was putting them [mentors and health professionals] together. They all did the same thing. That is to say, answer my questions and enlighten me on the various aspects of reduced mobility. On both sides, I would say that they did a lot on the same job. For me the plus side is that it [the mentor] brings sports into our exchanges.” Patients' confusion in these roles might impede their participation in the peer mentorship program because they might not perceive the benefits of engaging with mentors.

## Discussion

The purpose of this study was to identify barriers and facilitators to the delivery of the peer mentorship program provided by a community-based SCI organization at a rehabilitation center and characterize the collaboration processes between the community organizational staff and the health professionals. We gathered multiple perspectives from the individuals directly involved in the program, including peer mentorship program director, peer mentors, mentees, and health professionals. In addition to the barriers, facilitators, and collaboration processes identified, our study highlighted multiple strategies that the rehabilitation center and the community-based organization have taken to address/leverage these barriers/facilitators. The strategies may inform collaborative processes needed to establish a partnership between rehabilitation institutions and community-based organizations as per this peer mentorship program.

In alignment with previous studies using the CFIR in a healthcare context (28, 29), the barriers and facilitators identified in this study were found to influence the delivery of the peer mentorship program, including adaptability, cost, relative advantage, knowledge and beliefs about the intervention, culture, networks and communication, patient needs and resources, engaging, and reflecting and evaluating. The constructs identified covered all five CFIR domains and demonstrated a full breadth of the results. For example, the collaborative networks and communication between the mentors and the health professionals were found to be key for the program, which aligns with past research as being one of the most frequently used CFIR constructs (29). Our results enrich the literature by identifying how peer mentors and health professionals strengthen their network and communication. For instance, sharing workspace, having informal conversations, and maintaining clear professional boundaries were strategies used by the mentors and the health professionals within the current peer mentorship program. Future peer mentorship programs should consider these collaboration and networking strategies to ensure the success of program implementation.

Peer mentorship programs within rehabilitation settings often target specific health outcomes for people with SCI (e.g., self-efficacy) (9), while the program in our study had an objective of promoting broader outcomes such as social and community re-integration. The community-based organization's objective closely aligns with the health professionals’ goal of facilitating patients' transition from rehabilitation to community. This alignment has contributed to the consistent engagement in the program for both organizations. Creating a shared vision is important for organizations to work together, whereas it is often challenging (30). Carrying out group activities that can encourage staff members to openly share their perspectives may help organizations develop a shared vision (31). These group activities may also allow staff from community-based organizations and rehabilitation institutions to share decision-making in defining goals and structure of the peer mentorship program prior to its delivery.

Communication among staff members tends to be an important aspect to successful implementation of services/programs across healthcare contexts (32, 33). Similarly, the “networks & communication” construct was identified as a facilitator in our study as the relationship between the mentors and the health professionals was highly collaborative and interactive. Other rehabilitation institutions might experience more challenges in maintaining constant team communication, particularly for those with a larger team or a culture with low expectations for communication (34). Within the current program, an office space for mentors embedded into the rehabilitation center enhances communication between mentors and rehabilitation staff. When a physical space is not possible, leaders of community-based organizations and rehabilitation institutions should foster communication among staff members by encouraging team discussions, forming a coalition/learning group, and/or identifying an opinion leader who can oversee the implementation (35).

Interprofessional collaboration has received growing attention in healthcare (maybe add a reference here as an example?). In our study, “networks and communication” and “professional boundaries” were the two largest constructs identified in terms of data volume. The interprofessional relationship between the mentors and the health professionals was interpreted as a key aspect of program implementation and maintenance. In alignment with previous research, the interprofessional collaboration between the mentors and the health professionals has resulted in multiple benefits, including personal and professional growth, as well as good work efficiency (36, 37). Additionally, because the community-based organization prioritizes delivering peer mentorship in-person, the health professionals were able to help the mentors build connections with patients and create mentor-mentee meeting opportunities. Therefore, community-based organizations and rehabilitation institutions should enhance their staff members' skills in interprofessional collaboration in order to strengthen collaborations at an organizational level, including how to maintain professional boundaries (38, 39). Although establishing and maintaining professional boundaries is often challenging in healthcare practice (38, 40), the roles of peer mentors and health professionals appeared to be well defined by the formal agreement within the current peer mentorship program. Clear boundaries can ensure the quality of mentor-mentee relationships and mentors' well-being in a long term (6, 41).

Another important collaboration process that can be challenging for community-based organizations was engaging and building buy-in among health professionals prior to the program delivery. Frequent staff change can hinder the process of engaging health professionals in the peer mentorship program (42). To address these barriers, community-based organizations can identify a local “champion”/“opinion leader” who can influence health professionals' attitudes and beliefs (43, 44) Furthermore, program evaluation over time is deemed to be necessary for delivery sustainability. For programs in the early phase, assessment can focus on appropriateness, feasibility, and accessibility (45). For long-standing programs, tracking the impacts on people with SCI may be a priority (7).

### Limitations

First, we were only able to recruit two health professionals from the rehabilitation center during the COVID-19 pandemic, although there might be multiple other health professionals who were directly or indirectly involved in the peer mentorship program. The two recruited health professionals might have a favorable opinion about the peer mentorship program as they consented to participate in this study. However, obtaining the perspectives from the peer mentorship program coordinator, the mentors, and the mentees still enabled us to capture a broad picture of individuals actively involved in the program. Another limitation was that we did not apply the updated version of CFIR (46) because we collected and analyzed the data prior to the publication. However, our application of the original version of CFIR was found to be appropriate given that the facilitators, barriers, and collaboration processes were identified based on the original CFIR. Initial coding was conducted by the first author (ZS) individually. Engaging co-authors to develop and critique the themes was conducted to strengthen to rigor of our analyses.

## Conclusions

Using the CFIR to guide the data collection and analysis, we identified multiple barriers, facilitators, and collaboration processes to delivering the peer mentorship program within the local rehabilitation center. Our results may help other community-based SCI organizations and rehabilitation institutions develop, maintain, and optimize peer mentorship programs in various rehabilitation settings. Community-based SCI organizations and rehabilitation institutions may enhance interprofessional collaborations between organizational staff (e.g., peer mentors) and health professionals by creating shared workspace, facilitating informal conversations, and establishing professional boundaries.

## Data Availability

The original contributions presented in the study are included in the article/**Supplementary Material**, further inquiries can be directed to the corresponding author.
