# Supplementary material for: Delivery of a community-based peer mentorship program for people with spinal cord injury at a rehabilitation center
Source: Front Rehabil Sci. 2023 Nov 29;4:1296505. doi: 10.3389/fresc.2023.1296505 (PMC10716281; doi:10.3389/fresc.2023.1296505)
Supplement: Supplementary file 1 [file Table1.docx]

Supplementary Material

Appendix A – Interview Guide

**Interview questions with mentors**

1. Tell me about your role in the peer mentorship program at the rehabilitation center.
   1. what do you do in relation to the program? Your daily routine?
2. How is the peer mentorship program currently implemented?
   1. what is the modality of mentor-mentee interactions? e.g., duration, frequency?
   2. who developed the program/why the program being implemented at the rehabilitation center/how/why the collaboration between the community-based SCI organization and the rehabilitation center was initiated?
      1. if you don’t know, who may know, any names?
   3. what is the process for deciding whether changes are needed to the program so that it works well at the rehabilitation center?
   4. What supports are available to help you deliver the program at the rehabilitation center?
   5. What kind of provincial, or national policies and/or financial factors influenced the decision to implement the program?
3. Who are the other persons involved in the peer mentorship program?
   1. Who are the hospital staff involved? Names and their positions? How do you work with them?
   2. Can you describe your working relationships with them? / What kinds of interactions do you have with them?
   3. When you need to get something done or to solve a problem, who are your “go-to” persons?
   4. Is there anyone who involved but currently left the program? Can you tell me about them?

4. How do you feel about the peer mentorship program at the rehabilitation center?

- 1. How well do you think the program meets or doesn’t meet the needs of the clients with SCI?
  2. How well do or do not the hospital staff/professionals support the program?
  3. How is the program meeting or not meeting your expectations, primarily based on how it is delivered and/or integrated within the rehabilitation center?

5. What are your lesson learned from delivering the peer mentorship program?

1. What recommendations do you have to hospitals and rehabilitation centers that aim to integrate a SCI peer mentorship program?

**Interview questions with mentees:**

1. Could you please introduce yourself?
   1. How long had you been receiving healthcare services at the rehabilitation center?
   2. How long have you been receiving peer mentorship from the community-based SCI organization?
   3. (If the participant has already left the rehabilitation center), are you still receiving peer mentorship or other services from the community-based SCI organization?
2. How did you interact with the mentors at the rehabilitation center?
   1. What type of conversation did you have with the mentor?
   2. How did you benefit from the mentorship?
3. What service did you receive from healthcare professionals at the rehabilitation center?
   1. Are the healthcare professionals involved in the mentorship program?
   2. If so, who are they and how are they involved?
4. Thinking of what the mentors do at the rehabilitation center, what are the differences and similarities between their role and health professionals’ roles?
   1. What are the complementary areas between health professionals and mentors? / How do they complement each other’s work? How well do or do not the health professionals support the program?
   2. What added value do the health professionals bring on top of the mentorship service?
   3. Is there any area where the services provided health professional vs mentors are in conflict? / (for example: If a mentor offers an advice, might a nurse offer something is opposite?)
5. How do you feel about the peer mentorship program at the rehabilitation center?
   1. How well do you think the program meets or doesn’t meet your needs?
   2. How can the program be improved?
   3. What recommendations do you have to hospitals and rehabilitation centers that want to offer peer mentorship to their clients with SCI?

**Interview questions with health professionals:**

1. Tell me about your role at the rehabilitation center,
2. How have you been involved in the SCI peer mentorship program?
   1. What do you do in relation to the program?

3. Who are the other persons involved in the peer mentorship program?

- 1. What kinds of interactions do you have with them? / How do you work with them?
  2. Who are the health professionals involved in the peer mentorship program? What positions they have?
     1. How do you work with them in supporting the peer mentorship program?

4. How is the peer mentorship program currently implemented at the rehabilitation center?

- 1. how the clients with SCI interact with the mentors from the community-based SCI organization? (e.g., where they meet, how often?)
  2. Who developed the program/why the program being implemented at the rehabilitation center/ how/why the collaboration between the rehabilitation center and the community-based SCI organization was initiated?
     1. if you don’t know, who may know, any names?
  3. What is the process for deciding whether changes are needed to the peer mentorship program so that it works well at the rehabilitation center?
  4. When you need to get something done or to solve a problem, what do you do / who are your “go-to” persons?
  5. What kind of provincial, or national policies and/or financial factors influenced the decision to deliver the program?
  6. Is there anyone who involved but currently left the program? Can you tell me about them?

5. How do you feel about the peer mentorship program at the rehabilitation center?

1. How well do you think the program meets or doesn’t meet the needs of the clients with SCI?
2. How well do you think the health professionals have supported the program?
3. How is the program meeting or not your expectations, based on how it is delivered at the rehabilitation center?

6. What are your lesson learned about working with the mentors?

1. What recommendations do you have to hospitals and rehabilitation centers that aim to integrate a peer mentorship program for their clients with SCI?

**Interview questions with program director:**

1. Tell me about your role at the community-based SCI organization.

- 1. How long have you been working for the community-based SCI organization?
  2. What are your main job duties at the community-based SCI organization?

2. What role are you taking in the peer mentorship program at the rehabilitation center?

3. How does the community-based SCI organization and the rehabilitation center to deliver the peer mentorship program?

- 1. who developed the program/why the program being implemented at the rehabilitation center/how/why the collaboration between the community-based SCI organization and the rehabilitation center was initiated?
  2. How did your team get involved in the rehabilitation center? (e.g., is there an official agreement?)
  3. How does the community-based SCI organization integrate the mentors into the rehabilitation center?
  4. What is the decision-making process if the program needs changes?
  5. How do you describe this collaboration between the community-based SCI organization and the rehabilitation center?

4. What are the factors/things that influence the peer mentorship program?

- 1. What barriers/challenges has the community-based SCI organization faced to deliver this peer mentorship program?
  2. What supports the role of mentors at the rehabilitation center?

5. What are your lesson learned from delivering the peer mentorship program?

- 1. What recommendations do you have to hospitals and rehabilitation centers that aim to integrate a SCI peer mentorship program?
